# Locally-adapted reproductive photoperiodism determines population vulnerability to climate change in burying beetles

**DOI:** 10.1038/s41467-020-15208-w

**Published:** 2020-03-13

**Authors:** Hsiang-Yu Tsai, Dustin R. Rubenstein, Yu-Meng Fan, Tzu-Neng Yuan, Bo-Fei Chen, Yezhong Tang, I-Ching Chen, Sheng-Feng Shen

**Affiliations:** 1 Biodiversity Research Center, Academia Sinica, Taipei, 115 Taiwan; 2 Institute of Ecology and Evolutionary Biology, College of Life Science, National Taiwan University, Taipei, 115 Taiwan; 3 Department of Ecology, Evolution and Environmental Biology and Center for Integrative Animal Behavior, Columbia University, New York, NY 10027 USA; 4 Chengdu Institute of Biology, Chinese Academy of Sciences, Chengdu, 61004 People’s Republic of China; 5 Department of Life Sciences, National Cheng Kung University, Tainan, 70101 Taiwan

**Keywords:** Climate-change ecology, Evolutionary ecology

## Abstract

Understanding how phenotypic traits vary among populations inhabiting different environments is critical for predicting a species’ vulnerability to climate change. Yet, little is known about the key functional traits that determine the distribution of populations and the main mechanisms—phenotypic plasticity vs. local adaptation—underlying intraspecific functional trait variation. Using the Asian burying beetle *Nicrophorus nepalensis*, we demonstrate that mountain ranges differing in elevation and latitude offer unique thermal environments in which two functional traits—thermal tolerance and reproductive photoperiodism—interact to shape breeding phenology. We show that populations on different mountain ranges maintain similar thermal tolerances, but differ in reproductive photoperiodism. Through common garden and reciprocal transplant experiments, we confirm that reproductive photoperiodism is locally adapted and not phenotypically plastic. Accordingly, year-round breeding populations on mountains of intermediate elevation are likely to be most susceptible to future warming because maladaptation occurs when beetles try to breed at warmer temperatures.

## Abstract (web summary)

Understanding whether intraspecific trait variation results from local adaptation or phenotypic plasticity is crucial to predict species responses to climate change. Here the authors investigate geographically distinct burying beetle populations, showing that photoperiodism is a locally adapted, not phenotypically plastic, trait.

## Introduction

Assessing species vulnerability to anthropogenic climate change is critical for conserving global biodiversity^1–3^. Most current methods for predicting how species will respond to climate change depend to a large extent on species distribution models that relate environmental variables to the distribution of species and predict habitat suitability under climate change scenarios using species-specific functional traits^4,5^. However, since populations and not species adapt to changing environmental conditions^6–8^, determining the mechanisms underlying intraspecific differences in functional trait values is crucial for assessing species vulnerability to climate change^5,9,10^.

Intraspecific trait variation can be the result of either (1) local adaptation, where resident genotypes in each population would have on average a higher relative fitness in their local habitat than genotypes originating from other habitats, or (2) phenotypic plasticity, where a given genotype can produce different phenotypes in response to distinct environmental conditions^6^. Each of these mechanisms of trait variation is likely to influence the niche spaces of different populations in very different ways. For example, a widely distributed species can comprise populations of individuals with either wide niche breadths that can plastically respond to changes in environmental conditions^11,12^, or alternatively, narrow niche breadths that are locally adapted to specific environmental conditions^13,14^. Although populations that exhibit phenotypic plasticity may have the capacity to cope with climate change, the existence of locally adapted populations suggests genetically based geographic variation in traits^15,16^ that may not be able to respond as rapidly to changing climates^17^. However, distinguishing between phenotypic plasticity and local adaptation as potential mechanism underlying the capacity to cope with environmental change must be done empirically and experimentally, in order to better understand how mechanisms of trait variation influence population and species vulnerability to anthropogenic climate change.

The life histories of local populations (e.g., the optimal times to reproduce, migrate, or hibernate) often differ as biogeographic conditions vary^18–20^. Reproductive photoperiodism and thermal tolerance are two key life history, or functional, traits that help many organisms keep pace with warming environments, but differ in their degree of evolvability^21^. Variation in photoperiodism has largely been examined across broadscale latitudinal clines and constitutes a key evolutionary response to climate change^21–24^. In contrast, thermal tolerance appears to be a relatively conserved trait with limited evolvability^25,26^. The mechanisms underlying how different populations track the suitability of environments for breeding, particularly for widely distributed species with many isolated populations, remain largely unknown.

**Fig. 1:**
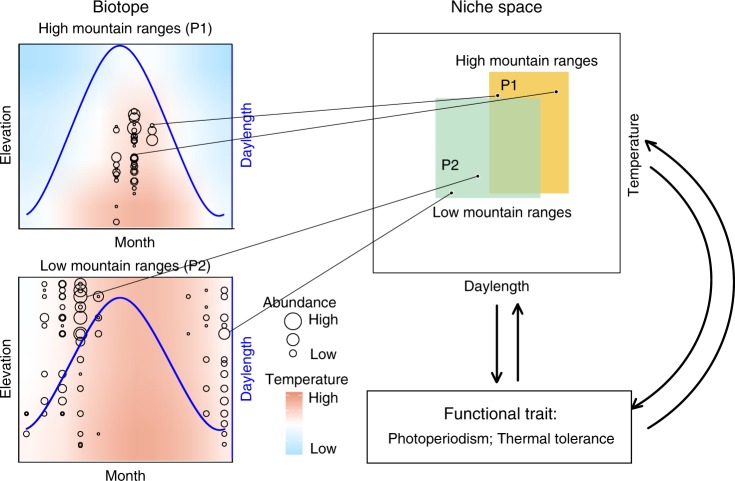
Illustration of the concept of niche-biotope duality for beetle populations on high and low mountain ranges. The geographical biotope is depicted by two dimensions, month and elevation, which correspond to the niche space of daylength (blue line) and temperature (the blue to red heatmap). The size of the open circles represents the abundance at each elevation in a given month. The black lines connect representative beetle abundances in high mountain ranges (P1) and low mountain ranges (P2) at different months and elevations of the biotope, with their corresponding points in the niches of the two populations. The yellow and green rectangles represent the size of the niche spaces of P1 and P2 along daylength and temperature gradients, respectively. The biotope figures are based on data from Wulai for the low mountain range and Mt. Jiajin for the high mountain range. Functional traits (indicated by arrows) represent the traits used to determine if they correspond to different niche spaces (i.e., daylength and temperature) occupied by different populations. Our figure is based on Figure 101 of Hutchinson^29^ and Figure 1 of Colwell and Rangel^27^.

Here, using a widely distributed species—the Asian burying beetle *Nicrophorus nepalensis—*we demonstrate that mountain ranges of differing maximum elevations and at varying latitudes offer distinct thermal niches, where thermal tolerance and reproductive photoperiodism interact to shape local adaptation of breeding phenology in the absence of phenotypic plasticity. We employ Hutchinson’s duality framework to distinguish between niche space (i.e., hyperspace with permissive conditions and requisite resources) and biotope (i.e., the current and future environmental conditions or “physical world” in biogeography)^27–29^, which enables the reciprocal projection between the geographic and temporal distribution of a population and its niche space (Fig. 1). Based on this reciprocal projection method, we use the spatiotemporal pattern of population abundances to estimate thermal and temporal niche spaces for five geographically distinct beetle populations, ultimately identifying photoperiodism as the key functional trait for population growth^21,24,30^, as it occupies different niche spaces among populations. We then use a lab common garden experiment to show that the local adaptation in reproductive photoperiodism—and not thermal plasticity—underlies intraspecific variation in reproductive phenology, followed by a reciprocal field transplant experiment to demonstrate that locally adapted traits confer higher fitness in a population’s native mountain range, but lower fitness in its non-native mountain range. Finally, after showing that reproductive photoperiodism is a heritable trait that is locally adapted to the environment each geographically distinct population experiences, we project future spatiotemporal distributions onto the physical world under different global warming scenarios, considering the capacity of shifting breeding phenology to estimate vulnerability to climate change in the absence of phenotypic plasticity.

## Results

### Empirical identification of thermal niche space in the field and lab

**Fig. 2:**
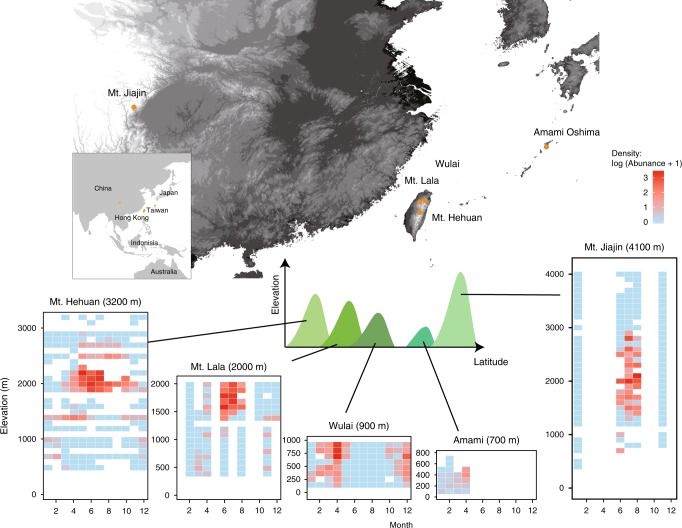
Map depicting the mountain ranges sampled and heatmaps of the spatial–temporal distributions of population density along the elevational gradient at each location. Abundance values were log-transformed according to the formula log (amount + 1). The color scale indicates the difference in the abundance of beetles at the five mountain ranges. The maximum elevation of each mountain range (indicated on the map by orange circles) is given in parentheses. The numbers of the beetles collected in total: Mt. Hehuan: *n* =1791; Mt. Lala: *n* = 347; Wulai: *n* = 498; Amami: *n* = 144 ; and Mt. Jiajin: *n* = 517 . Maps were created based on the silhouettes from Farr et al.^69^.

**Fig. 3:**
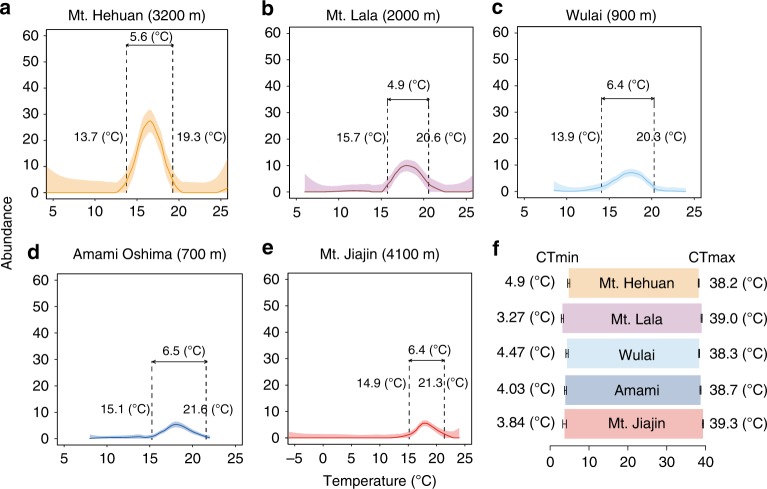
Burying beetle abundances along the elevational temperature gradients, and their thermal niche spaces and thermal tolerance ranges for each of the five mountain ranges. The thermal niches of the **a** Mt. Hehuan, **b** Mt. Lala, **c** Wulai, **d** Amami Oshima, and **e** Mt. Jiajin populations, the line and polygon areas are fitted trend and confidence intervals from the non-parametric regression (LOESS), and **f** the thermal tolerance ranges, defined as CTmax minus CTmin, of five populations. The standard error was presented as the error bar. The maximum elevation of each mountain range is given in parentheses.

We began by investigating the spatiotemporal distribution of *N. nepalensis* populations across a variety of mountain ranges and latitudes in Asia (mainland China, Taiwan, and Japan) to derive the thermal niche space of different populations (Fig. 2). Within each mountain range, we depicted the spatiotemporal distribution of burying beetles by quantifying population densities along elevational gradients each month (Fig. 2). We then derived a LOESS curve relationship between population density and the corresponding temperature at each elevation to estimate the thermal niche space of each population, assuming that higher densities represent fitter populations (Fig. 3a–e). We found that the upper limits of the population thermal niche range from 18.8 to 21.1 °C and increase slightly with increasing latitude, whereas the lower limits of the population thermal niche range from 14.7 to 16.5 °C and do not vary with latitude. Moreover, laboratory experiments demonstrated that the beetle’s upper thermal limit was largely similar among populations, with the critical thermal maximum (CTmax) temperature ranging from 38.2 to 39.0 °C (Wulai: *n* = 56; Amami: *n* = 40; Mt. Hehuan: *n* = 154; Mt. Lala: *n* = 37; and Mt. Jiajin: *n* = 20), and the critical thermal minimum (CTmin) temperature ranging from 3.27 to 4.47 °C (Wulai: *n* = 64; Amami: *n* = 43; Mt. Hehuan: *n* = 65; Mt. Lala: *n* = 23; and Mt. Jiajin: *n* = 20; Fig. 3f). Thus, the five populations of *N. nepalensis* on mountain ranges of different maximum elevations and at different latitudes exhibit only minor differences in their thermal niche space and their thermal limits.

### Accurate projection of temporal niche space from thermal niche space

**Fig. 4:**
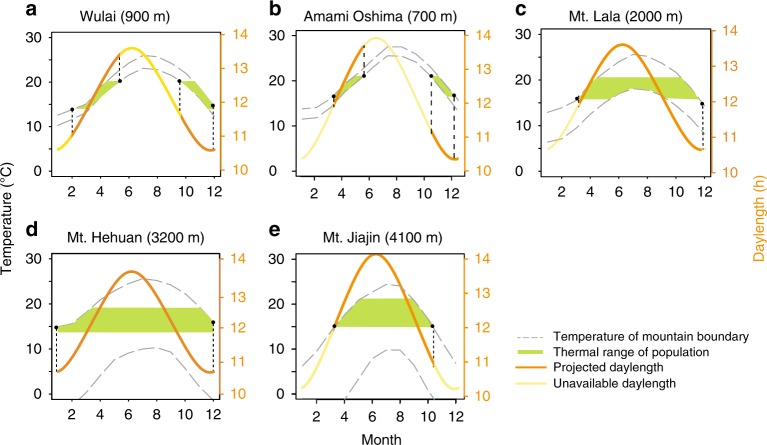
The temperature and corresponding photoperiod in relation to elevation, month, and daylength for each of the five mountain ranges. The thermal niche space where beetles are present, estimated in Fig. 3, (filled green regions) and the environmental temperature at different elevations (the gray dash line) for the different mountain ranges determine the suitable daylengths (orange lines) and unavailable daylengths (yellow lines) of the beetles occurrence in **a** Wulai, **b** Amami Oshima, **c** Mt. Lala, **d** Mt. Hehuan, and **e** Mt. Jiajin. The maximum elevation of each mountain range is given in parentheses.

Given the broadly similar thermal niches and thermal limits among populations identified in our field and lab experiments, we further investigated how temporal niche space might select for other functional traits, enabling *N. nepalensis* to persist in similarly favorable thermal environments. Specifically, we used the thermal niche space of each population that we quantified from our field surveys to project back to the physical environment (i.e., biotope) of each mountain range (data from WorldClim^31^), in order to investigate each population’s temporal niche space, which is represented by the daylength that is suitable for breeding (Fig. 4). Our projections predict that both maximum elevation and latitude of a mountain range will play a critical role in shaping a populations’ temporal niche space, which, in turn, influences the breeding phenology of each population. Breeding phenology is represented by reproductive photoperiodism, or the reproductive activity in certain seasons based on photoperiodic cues. Specifically, the low mountain ranges of Wulai (maximum 900 m) and Amami Oshima (maximum 700 m) are predicted to be reproductively active only in the spring when daylength is shorter (Fig. 4a, b), whereas populations from the high mountain ranges of Mt. Hehuan (maximum 3200 m) and Mt. Lala (maximum 2000 m) should be reproductively active over most of the year—including under both long- and short-day conditions (Fig. 4c, d). However, the high mountain range population from Mt. Jiajin (maximum 4100 m) should only breed during summer when daylength is longer because of the colder winters associated with its higher latitude (Fig. 4e). Thus, our projection based on thermal niche space suggests that there is likely to be substantial variation in temporal niche space (relative to thermal niche space), and therefore breeding phenology and reproductive photoperiodism in *N. nepalensis*.

Next, we compared the predicted temporal niche space (Fig. 4) with the actual breeding phenology derived from empirical population density data (Fig. 2). We found that, for the three populations with year-round data (Wulai, Mt. Lala, and Mt. Hehuan), the predicted temporal niche space matched well with the empirical data. The predicted breeding season of the low mountain range Wulai population was from October to May with the exception of January, which was too cold for the beetles to be active. The actual population density data indeed showed that October to May was the breeding season, although there were still scattered insects around in January, suggesting at least some breeding during this month. Similarly, for the high mountain range populations of Mt. Hehuan and Mt. Lala, the actual population density data were in nearly complete agreement with the predicted pattern (Figs. 2 and 4). We note that although we do not have January data from Mt. Lala, because there were no beetles around in February and January was even colder than February, it is unlikely that there were beetles breeding in January, as predicted.

### Common garden experiment of reproductive photoperiodism

**Fig. 5:**
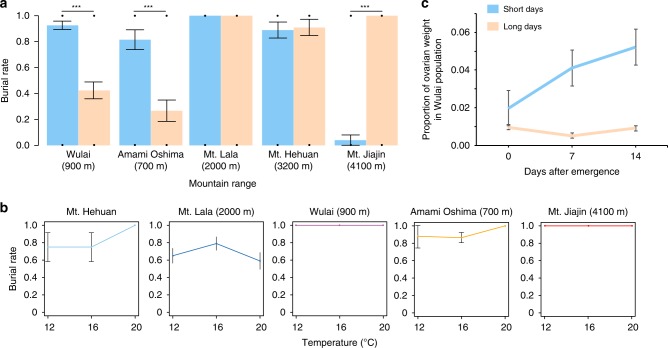
Results of common garden experiment examining reproductive photoperiodism in five beetle populations. **a** Breeding likelihood in terms of burial rates under short- (blue bar) and long-days (orange bar; Wulai: *χ*²_1_ = 39.93, *p* < 0.001, *n* = 126, Amami Oshima: *χ*²_1_ = 18.19, *p* < 0.001, *n* = 57, Mt. Hehuan, *χ*²_1_ = 0.08, *p* = 0.78, *n* = 50, Mt. Lala: *χ*²_1_ = 0, *p* = 1, *n* = 32, and Mt. Jiajin: *χ*²_1_ = 59.51, *p* < 0.001, *n* = 49; post hoc comparison: Wulai: *z* = −5.46, *P* < 0.0001, Amami Oshima: *z* = −3.755, *P* = 0.0002, Mt. Hehuan: *z* = −0.253, *P* = 0.8, Mt. Lala: *z* = −0.981, *P* = 0.327, and Mt. Jiajin: *z* = 4.338, *P* < 0.0001). **b** Breeding likelihoods under three temperature conditions (12, 16, and 20 °C; Wulai: *χ*²_2_ = 3.63, *p* = 0.16, *n* = 24; Amami Oshima: *χ*²_2_ = 2.84, *p* = 0.24, *n* = 88; Mt. Lala: *χ*²_2_ = 0, *p* = 1, *n* = 44, Mt. Hehuan: *χ*²_2_ = 5.33, *p* = 0.07, *n* = 70; and Mt. Jiajin: *χ*²_2_ = 0, *p* = 1, *n* = 18). **c** Ovarian development in the Wulai population after emergence under short- (blue line) and long-days (orange line). The maximum elevation of each mountain range is given in parentheses (short-days: *χ*²_2_ = 29.25, *p* < 0.001, *n* = 55, long-days: *χ*²_3_ = 0.35, *p* = 0.95, *n* = 44; post hoc comparison:). All the analyses were conducted by two-sided GLM, and the *p* values were obtained from the two-sided tests. The standard error was presented as the error bar; *p* < 0.1; *p < 0.05; **p < 0.01; ***p < 0.001.

We performed a common garden experiment in the lab to (1) experimentally test the temporal niche space projections (and patterns of reproductive photoperiodism), generated from our thermal niche space estimates, and then (2) investigate the mechanism (local adaptation vs. phenotypic plasticity) underlying intraspecific functional trait variation in reproductive photoperiodism in *N. nepalensis*. Consistent with the temporal niche space projections, our common garden experiment showed that beetle strains originating from the low mountain ranges of Wulai and Amami Oshima had higher likelihoods of breeding (i.e., higher carcass burial rates) in short- (light:dark = 10:14 h) than long-day treatments (light:dark = 14:10 h), confirming that these populations breed primarily in winter (Wulai: general linear model (GLM), *χ*²_1_ = 39.93, *p* < 0.001, *n* = 126; and Amami Oshima: GLM, *χ*²_1_ = 18.19, *p* < 0.001, *n* = 57). In contrast, beetle strains originating from the high mountain ranges of Mt. Hehuan and Mt. Lala had equally high likelihoods of breeding under both long- and short-days, confirming that they are year-round breeders (Mt. Hehuan: GLM, *χ*²_1_ = 0.08, *p* = 0.78, *n* = 50; and Mt. Lala: GLM, *χ*²_1_ = 0, *p* = 1, *n* = 32). Moreover, these high mountain range populations are comprised of year-round breeders (i.e., individuals that can breed in both long- and short-days), not by several seasonally isolated subpopulations. Finally, the high latitude and high mountain range beetle strain from Mt. Jiajin had a much higher likelihood of breeding in long- than short-days, confirming that they can only breed in summer (GLM, *χ*²_1_ = 59.51, *p* < 0.001, *n* = 49; Fig. 5a). Consistent with the idea of local adaptation of reproductive photoperiodism but not thermal tolerance, our common garden experiment demonstrated that there were no significant differences in breeding temperature (i.e., the temperature that beetles attempted to breed) among all five populations (Fig. 5b, Wulai: GLM, *χ*²_2_ = 3.63, *p* = 0.16, *n* = 24, Amami Oshima: GLM, *χ*²_2_ = 2.84, *p* = 0.24, *n* = 88, Mt. Lala: GLM, *χ*²_2_ = 0, *p* = 1, *n* = 44, Mt. Hehuan: GLM, *χ*²_2_ = 5.33, *p* = 0.07, *n* = 70, and Mt. Jiajin: GLM, *χ*²_2_ = 0, *p* = 1, *n* = 18). Thus, variation in breeding phenology among *N. nepalensis* populations is primarily due to locally adapted heritable differences in reproductive photoperiodism and not plastic responses to local thermal conditions.

A crucial component of achieving optimal timing for breeding is that organisms need to be able to physiologically prepare for the arrival of the suitable season. To determine whether beetle preparation for breeding is based on photoperiodic control, we examined ovarian development in the Wulai low mountain range population strain raised under both long- and short-days. We found that female ovaries only developed under short- (GLM, *χ*²_2_ = 29.25, *p* < 0.001, *n* = 55) not long-days (GLM, *χ*²_3_ = 0.35, *p* = 0.95, *n* = 44; Fig. 5c), again confirming that breeding phenology in *N. nepalensis* is dependent upon photoperiodic cues. Together with the results of the common garden experiment, our work suggests that reproductive photoperiodism is a key functional trait influencing breeding phenology and the temporal niche space of *N. nepalensis*.

### Reciprocal transplant experiment of reproductive photoperiodism

**Fig. 6:**
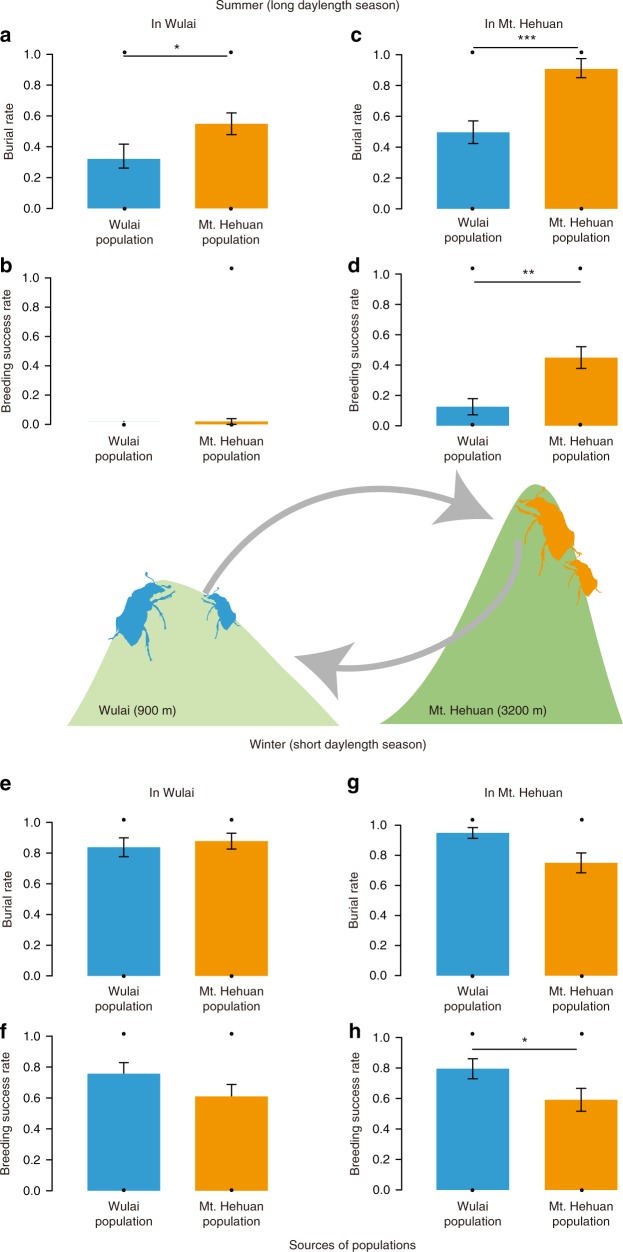
Results of reciprocal transplant experiment between the low elevation Wulai and high elevation Mt. Hehuan populations in winter and summer. Breeding likelihood and breeding success of the Mt. Hehuan and Wulai populations in summer in Wulai **a**, **b** (burial rate: *χ*²_1_ = 4.60, *p* = 0.03, *n* = 120, breeding success: *χ*²_1_ = 1.13, *p* = 0.29, *n* = 120; post hoc comparison: burial rate: *z* = 2.202, *P* = 0.028, breeding success: *z* = −0.955, *P* = 0.34), and Mt. Hehuan **c**, **d** (burial rate: *χ*²_1_ = 12.96, *p* < 0.001, *n* = 93; breeding success: *χ*²_1_ = 8.60, *p* = 0.003, *n* = 93; post hoc comparison: burial rate: *z* = 4.185, *P* < 0.0001, breeding success: *z* = 3.012, *P* = 0.026), and in winter in Wulai **e**, **f** (burial rate: *χ*²_1_ = 0.26, *p* = 0.61, *n* = 78; breeding success: *χ*²_1_ = 1.90, *p* = 0.17, *n* = 78), and Mt. Hehuan **g**, **h** (burial rate: *χ*²_1_ = 2.45, *p* = 0.12, *n* = 83; breeding success: *χ*²_1_ = 3.88, *p* = 0.05, *n* = 83; post hoc comparison: burial rate: *z* = −1.969, *P* = 0.049). All the analyses were conducted by GLMM, and the *p* values were obtained from the two-sided tests. The standard error was presented as the error bar; *p* < 0.1; *p < 0.05; **p < 0.01; ***p < 0.001.

To determine whether the locally adapted variation in reproductive photoperiodism is in fact adaptive, we performed a reciprocal transplant experiment between the high mountain range population of Mt. Hehuan and the low mountain range population of Wulai during both summer and winter, and then measured the beetles’ fitness in terms of breeding likelihood and breeding success (Fig. 6, sensu ref. ^32^). During summer, we found a significant interaction between mountain range and the source population being transplanted. Specifically, although Mt. Hehuan individuals transplanted to Wulai in summer had a higher likelihood of breeding than native Wulai individuals (generalized linear mixed model, GLMM, *χ*²_1_ = 4.60, *p* = 0.03, *n* = 120; Fig. 6a), both populations failed to breed successfully (GLMM, *χ*²1 = 1.13, *p* = 0.29, *n* = 120; Fig. 6b) because either their eggs did not hatch under such high temperatures or they did not even attempt to breed. Despite overall low reproductive success, these results suggest that individuals from the Wulai population may have had higher fitness than those from the Mt. Hehuan population in summer at Wulai because they did not waste energy attempting to breed under unfavorably hot conditions (mean temperature is 24.0 °C in summer of Wulai). This temperature is much higher than the average temperatures, which are 18 °C and 13 °C, respectively, during the breeding seasons of Mt. Hehuan and Wulai. In the reciprocal transplant (Mt. Hehuan in the summer), we found that native Mt. Hehuan individuals had a higher likelihood of breeding and greater breeding success than transplanted Wulai individuals (GLMM, burial rate: *χ*²_1_ = 12.96, *p* < 0.001, *n* = 93; Fig. 6c; breeding success: *χ*²_1_ = 8.60, *p* = 0.003, *n* = 93; Fig. 6d). These results also suggest that the Mt. Hehuan population has higher fitness locally than the Wulai population. During winter, however, the two populations showed no difference in the likelihood of breeding at either mountain range (GLMM, Wulai: burial rate: *χ*²_1_ = 0.26, *p* = 0.61, *n* = 78; Fig. 6e; Mt. Hehuan: burial rate: *χ*²_1_ = 2.45, *p* = 0.12, *n* = 83; Fig. 6g). Yet, while there was also no difference in breeding success between two populations at Wulai (GLMM, *χ*²_1_ = 1.90, *p* = 0.17, *n* = 78; Fig. 6f), the Wulai population had marginally higher breeding success than the Mt. Hehuan population at Mt. Hehuan during winter (GLMM, *χ*²_1_ = 3.88, *p* = 0.05, *n* = 83; Fig. 6h). Together, these experiments suggest that the year-round breeding of the Mt. Hehuan population and the short-day breeding of the Wulai population are both locally adapted traits that confer higher fitness for each population in its native mountain range, but lower fitness in its non-native mountain range.

### Predicting responses under future climate change

Despite it being a widely distributed species, inhabiting a locally adapted niche space may have a profound effect on *N. nepalensis’* vulnerability to future warming because niche space determines a population’s ability to shift its spatial and temporal distribution (i.e., future biotope) under climate warming. To understand how future climate change might influence populations on all five mountain ranges, we used Hutchinson’s duality framework^27–29^ to explore population vulnerability based on thermal and temporal niche characteristics under a scenario of Greenhouse Gas Representative Concentration Pathway (RCP) 8.5 for 2081–2100 adopted by the Intergovernmental Panel on Climate Change for its fifth Assessment Report^33^.

**Fig. 7:**
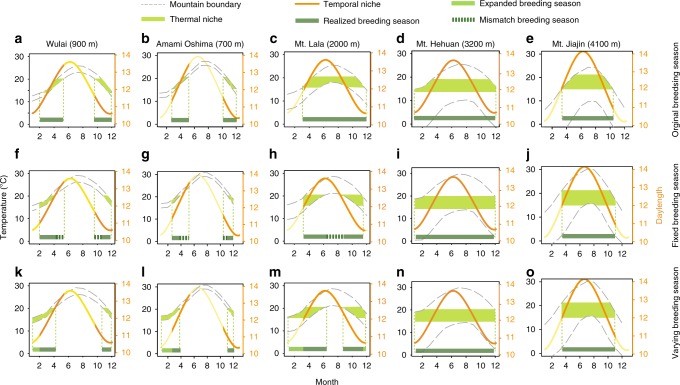
Range and phenology simulation results under the RCP 8.5 warming scenario. **a**–**e** Current phenology, the thermal niche space of beetles’ presence, estimated in Fig. 3, (filled green regions; see also Fig. 4) and the environmental temperature in the elevational range at each mountain (the gray dash line) determine each beetle population’s temporal niche (orange lines). Global warming may lead to a mismatch (green dot lines) or shifted phenology (light green lines). **f**–**j** Predicted breeding season under the fixed phenology assumption. **k**–**o** Predicted breeding season under varying phenology assumption. The maximum elevation of each mountain range is given in parentheses.

We first explored a scenario where populations do not shift their breeding phenology (fixed breeding season, Fig. 7f–j, compared with the current phenology Fig. 7a–e). Under this scenario, the low mountain range populations of Wulai and Amami Oshima, as well as the lower of the high mountain range population of Mt. Lala (maximum 2000 m), would be predicted to face a high risk of extinction because of the mismatch between suitable thermal conditions and breeding season (Fig. 7f, g). In contrast, thermal conditions during the breeding season of the other two high mountain ranges of Mt. Hehuan and Mt. Jiajin are predicted to be less impacted by global warming (Fig. 7i, j). However, if we assume that shifting breeding phenology is possible, based only on their niche characteristics, populations of short-day breeders at Wulai and Amami Oshima will be less affected by warming, as they are more likely to advance breeding in winter (Fig. 7k, l). Although the population of Mt. Lala is likely to extend its breeding season to winter, the hot summer will still disrupt breeding activity (Fig. 7m). These opposing effects—increasing length of the breeding season but disruption of breeding in summer—will likely differentially impact population size and vulnerability. Finally, populations from the high mountain ranges of Mt. Hehuan and Mt Jiajin will be less impacted by climate change, since suitable breeding temperatures will still be available at higher elevations (Fig. 7n, o). Thus, year-round breeding populations on mountains of intermediate elevation are likely to be most susceptible to future warming because maladaptation is likely to occur when beetles try to breed at warmer temperatures.

## Discussion

Identifying the key functional traits that underlie animal distributions has proven challenging. By employing Hutchinson’s duality framework^27–29^, we show that the key niche space difference among populations of burying beetles lies primarily along the daylength axis, and only to a much lesser degree along the temperature axis. Accordingly, reproductive photoperiodism is the primary functional trait responsible for the differential temporal distribution of breeding phenologies among populations (Fig. 4). We further demonstrate using a common garden experiment that variation in breeding phenology among different populations is shaped by local adaptation in reproductive photoperiodism and not thermal plasticity. Therefore, the widely distributed burying beetle *N. nepalensis* contains many populations across Asian mountain ranges with locally adapted breeding phenology, each exhibiting different forms of reproductive photoperiodism that our reciprocal transplant experiment confirmed maximizes fitness. Thus, our study provides empirical and experimental evidence consistent with increasing calls for examining the mechanisms underlying variation in key climate-related functional traits at the population—not just species—level in order to understand organismal vulnerability to climate change^6–8^.

Our study supports the idea that shifts in breeding phenology should precede shifts in traits related to thermal tolerance in seasonal environments^21^. This is because adaptive changes in breeding phenology are presumably physiologically more feasible than evolutionary changes in thermal tolerance when facing different environments^26,34,35^. Importantly, fitness in seasonal environments depends not only on the optimal time to engage in critical activities like breeding, hibernation, or aestivation, but also on the ability to forecast and prepare for changing seasons before they arrive^20,36^. Although a number of studies have demonstrated that breeding phenology has changed due to climate warming at the species level^37–40^, many fewer have focused on trait variation at the population level; those that have done so have largely compared thermal tolerances rather than phenological traits^41,42^. A rare exception comes from a longitudinal study of pitcher-plant mosquitos showing that populations shifted toward shorter daylengths as growing seasons have become longer, especially in temperate regions^30^. Our study differs from the mosquito study in that we compare several contemporary populations at different mountain ranges that vary in both elevation and latitude. Our simulation shows that climate warming can have diverse effects on the breeding seasons of insects, which can lead to longer, unchanged, or disrupted breeding seasons (Fig. 7), which is different from the longer breeding season effect found in the study of pitcher-plant mosquitos in temperate regions^30^.

In summary, research on species vulnerability to climate change has largely focused on how thermal tolerance will influence ranges sizes (e.g., species distribution modeling) without considering the capacity of shifting breeding phenology, something that is often regulated by photoperiodism^43,44^. Although phenotypic plasticity is generally considered critical for climate change adaptation, our study suggests that locally adapted reproductive photoperiodism may also facilitate phenological shifts and thus mitigate fitness loss under global warming. However, local adaptation can be deleterious if breeding phenology becomes disrupted due to a mismatch between elevated temperature and reproductive photoperiodism.

More generally, understanding the relationship and pattern of mismatch between current niche space and future biotope will be crucial for inferring the vulnerability of populations under climate change. Accounting for intraspecific trait variation and its genetic basis allows for a more mechanistic understanding of how populations respond differently to climate change and how these changes may influence abundance and distribution at the species level^8,45,46^. For widely distributed species that occur at many locally adapted sites, climate change is likely to impact populations differently and not simply affect a species as a whole. Such impacts are often overlooked because researchers fail to use an experimental approach to identify signatures of local adaptation, instead assuming that species typically vary only because of phenotypic plasticity. Therefore, integrating experimental approaches to define niche limits with trait-based distribution models will provide a more rigorous way to assess population and species vulnerability^47–50^. Ultimately, conserving locally adapted populations and their underlying genetic diversity may be essential for species survival in the face of future climate change, particularly for those species that do not exhibit phenotypic plasticity in key functional traits^51,52^.

## Methods

### Study species

Burying beetles use small vertebrate carcasses to reproduce and as a food source for themselves and their offspring^53–55^. Previous research in the lab has shown that *Nicrophorus* beetles can reproduce at least three times over the course of their lifetimes^56^. Laboratory breeding experiments have also shown that when burying beetles reach a carcass, they mate, take ~3–4 days to remove the fur or skin, and smear the carcass with the liquid secreted by the mouth and rectum to delay decomposition^57^. Next, they make the carcass into a “meatball”, bury it under the soil, and eventually lay eggs near the carcass^58^. Our observations indicate that the larvae, which have a total of three-instar stages, hatch ~1 day after egg laying, at which time they begin to take up the nutrients of the meatball (H.-Y.T., Y.-M.F., T.-N.Y., B.-F.C., and S.-F.S., unpublished data). Two weeks after burial, the larvae grow into three-instar larvae that are ready to leave the nest for pupation. In total, the larvae take 1.5 months to emerge as adults (H.-Y.T., Y.-M.F., T.-N.Y., B.-F.C., and S.-F. S., unpublished data). The average age of the beetles in our experiments was 135 days after the larvae leave the meatball for pupation, and there was no age difference between the sexes (H.-Y.T., Y.-M.F., T.-N.Y., B.-F.C., and S.-F.S. unpublished data).

### Study site

The burying beetle *Nicrophorus nepalensis* (Coleoptera: Silphidae) occurs widely across mountain ranges throughout Asia^59^. We quantified the monthly population densities of *N. nepalensis* at 100 m intervals along elevational gradients at five mountain ranges in Asia located from 24 °N to 30 °N that varied in maximum elevation from 700 m to 4100 m (Fig. 2). We define the breeding season of *N. nepalensis* as the months that at least one beetle appeared in the trap. The mountain ranges, which differed in both their thermal environment (due to elevation) and daylength (due to latitude), included two low mountain ranges—Wulai in Taiwan, with natural habitats ranging from 200 m (121° 51′ E, 24° 83′ N) to 900 m (121° 54′ E, 24° 85′ N) above sea level and Amami Oshima in Japan, ranging from 60 m (129° 27′ E, 28° 32′ N) to 700 m (129° 32′ E, 28° 30′ N)—and three high mountain ranges—Mt. Lala in Taiwan, ranging from 400 m (121° 51′ E, 24° 60′ N) to 2000 m (121° 43′ E, 24° 73′ N), Mt. Hehuan in Taiwan, ranging from 500 m (121° 00′ E, 23° 98′ N) to 3200 m (121° 27′ E, 24° 13′  N), and Mt. Jiajin in China, ranging from 800 m (102° 84′ E, 30° 23′ N) to 4100 m (102° 68′ E, 30° 86′ N).

### Density survey

To estimate beetle densities and determine breeding phenology at each site, we averaged the number of beetles by month because we replicated the experiments for ~3 years. Adult burying beetles were collected using hanging pitfall traps baited with rotting pork^32,60^ (mean ± SE: 100 ± 10 g) at Mt. Hehaun, Taiwan (January 2016 to May 2018), Mt. Lala, Taiwan (February–April, August, and November 2017; February, June, and July 2018), Wulai, Taiwan (January 2016 to May 2018), Amami Oshima, Japan (February 2015; April 2016; March–April 2018), and Mt. Jiajin, China (June–August 2017, January, June–August 2018, and November 2019; Fig. 1). The pitfall traps were always checked in the morning on the fourth day of the experiment. The air temperature at every site was measured using iButton® devices that were placed ~120 cm above the ground within a T-shaped PVC pipe to prevent direct exposure to the sun. Since carcass preparation and burial, which occur above ground, are critical to the successful breeding of burying beetles, we believe that air temperature is an appropriate measure of environmental temperature (see also refs. ^32,60,61^). Although we acknowledge that soil temperature could be important for the larva development, those data are not available.

We only brought two male and two female beetles from each plot back to the lab to ensure that we did not have a strong influence on the population density in the field. All wild-caught beetles were transported to the laboratory walk-in growth chambers and allowed to reproduce in captivity. Beetles captured in the short photoperiod season were kept in short-day conditions (10 h light: 14 h dark), and those captured in the long photoperiod season were kept in long-day conditions (14 h light: 10 h dark). The temperature and humidity in all of the walk-in growth chambers were set to the same conditions in both photoperiodic regimes (daily temperature cycles between 19 °C at noon and 13 °C at midnight; RH: 83–100%), which imitated natural conditions at 2100 m elevation on Mt. Hehaun. Beetles were housed individually in 320 ml transparent plastic cups and fed superworms (*Zophobas morio*) weekly if they were kept for more than three days before the experiment.

To conduct the experiments, we obtained permits required by local governments, forestry bureaus and national parks annually in Taiwan in 2014–2018, MOUs between two academic institutes required by the National Forestry and Grassland Administration in China, and the experimental permit required by the Ministry of Environment in Japan.

To bring the beetles collected abroad back to Taiwan, we  obtained the permit required by the Bureau of Animal and Plant Health Inspection and Quarantine in 2018 and 2019.

### Establishment of lab strains

We established lab strains from each population by pairing male and female beetles collected from each mountain range for the subsequent experiments. The generation of the initial lab strain was denoted as wild-type (WT). In the WT generation, we established at least 20 families, ~600 individuals in total, to maintain population structure in the lab. We used beetles collected in different hanging pitfall traps to ensure that founding beetles in the lab strains were unrelated to each other. We bred a pair of beetles in a 20 × 13 × 13 cm box with 10 cm of soil and a rat carcass (75 ± 7.5 g). The parents were allowed to remain in the breeding box until larvae dispersed to pupate (~2 weeks after introducing adult beetles). All dispersing larvae from each breeding box were collected and allocated to a small, individual pupation box. The larvae were evenly but arbitrarily divided and raised under two photoperiodic conditions, as described above.

### Temperature data

We used average monthly climate data to depict the temperature pattern at five mountain ranges based on WorldClim v2 (30 s spatial resolution; records from 1970 to 2000 (ref. ^31^)): http://worldclim.org/version2.

### Common garden experiment

Common garden experiments allow for a test of local adaptation vs. phenotypic plasticity. To observe how beetle reproductive behavior from each population changes in response to photoperiod, we conducted a solitary pairing experiment in two photoperiodic regimes: long- (10 h dark: 14 h light) and short-day conditions (14 h dark: 10 h light). The temperature and humidity were set to the same conditions in both photoperiodic regimes, as described above. All larvae from a pair of beetles were separated into the two photoperiodic conditions immediately at the time of dispersal. We used sexually mature beetles aged 2–3 weeks after emergence.

To observe how beetle reproductive behavior from each population changes in response to temperature, we conducted pair breeding experiments in three average temperature condition: 12 °C, 16 °C, and 20 °C. The humidity was set to the same conditions in all temperature conditions (RH: 83–100%). The photoperiod regime was set to the same as short-day conditions (14 h dark: 10 h light) for the Wulai, Amami Oshima, Mt. Lala, and Mt. Hehuan population, but to long-day conditions (10 h dark: 14 h light) for the Mt. Jiajin population since these beetles can only breed under long-day conditions. All larvae from a pair of beetles were separated into the two photoperiodic conditions immediately at the time of dispersal. We used sexually mature beetles aged 2–3 weeks after emergence.

We placed a male and a female pair with a rat carcass (75 ± 7.5 g) under both photoperiodic conditions in a transparent plastic container (21 × 13 × 13 cm with 10 cm of soil depth) and gave them 2 weeks to breed. If they did not bury the carcass by the 14th day after the experiment began, the pairs were moved to a new container with a new carcass under the same environmental conditions to repeat the experiment. A case in which the parents fully buried the carcass and had offspring within two trials was regarded as successful breeding attempt. A case in which the parents failed to bury a carcass, or they buried it but did not have offspring in two consecutive trials, was regarded as a failed breeding attempt. Each experiment was conducted with different pairs that thus are independent samples.

### Transplant experiment

Reciprocal transplant experiments allow for direct tests of local adaptation, whereby the fitness of the native population is compared to that of the non-native one. To this end, we conducted reciprocal transplant experiments between the low mountain range Wulai and high mountain Mt. Hehuan populations in January, February, December, and June, early August in 2016 and 2017 to compare winter and summer conditions. In each season, we chose the three sites on each mountain that the highest beetle abundance according to our field surveys. We conducted transplant breeding experiments in which lab strains from either population were transplanted to either non-native mountain range (i.e., the lab strain individuals originating from the Wulai population were transplanted to Mt. Hehuan, and vice versa) or to the native mountain range as a control (i.e., the lab strain individuals originating from the Wulai or Mt. Hehuan population were transplanted to Wulai or Mt. Hehuan, respectively).

In each trial, we placed a WT male and a WT female with a rat carcass (75 ± 7.5 g) in the breeding pot, which was covered by a gauze web and buried in the soil to keep the beetles inside the pot and prevent other insects from invading. Each breeding pot is 19 cm in length, which is deep enough for beetles to bury the carcass and lay eggs. A 75 g rat carcass was placed on the soil and covered with a 21 × 21 × 21 cm iron cage with a mesh size of 2 × 2 cm to prevent vertebrate scavengers from accessing the carcass^32,60^. We quantify both breeding likelihood and breeding success. Breeding likelihood, defined by the burial rate, is quantified by whether burying beetles bury the carcass (i.e., burial or non-burial). To quantify breeding success, we exhumed the carcasses ~14 days after they were buried and collected third instar larvae, if there were any. If the parents had offspring, we regarded it as a successful breeding event; if not, we regarded it as a failed breeding event. Each experiment was conducted with different pairs that thus are independent samples.

### Ovary dissection

To understand whether beetle preparation for breeding is based on photoperiodic control, we dissected and quantified ovarian weight at three-time points: on days 0, 7, and 14 after emergence. The general dissection protocol followed the method described by Wilson and Knollenberg^62^. Briefly, we placed the beetles on ice for 1 h to euthanize them. We then measured their body weight and cut down their abdomens. Ovaries were dissected into Ringer’s solution. Next, we removed the spermatheca and accessory glands and immediately quantified the wet weights of each organ.

### Thermal tolerance

To investigate burying beetles’ thermal tolerances—CTmax and CTmin—we tested each populations’ thermal tolerance range, following the protocol of Sheldon and Tewksbury^63^. Briefly, we measured each burying beetle’s pronotum, and then transferred them to separate glass cups (200 mL, with lid) at an initial temperature of 25 °C for 1 h to ensure beetle body temperatures were the same going into experimental tests. A 5 mm layer of peat soil was placed on the bottom of each cup to allow beetles to walk freely. Each cup containing a beetle was then submerged into either a 50 °C or −10 °C water bath to test its upper or lower thermal limit. We chose 50 °C as the warm trial temperature because it made beetles achieve the upper thermal limit efficiently before desiccation. We chose −10 °C as the cold trial temperature because we found that this temperature did not immediately lead to the loss of righting for the beetles (but colder temperatures did). Since a layer of peat soil was placed at the bottom of the cup, the actual soil temperatures that a beetle experienced in the cup were ~42 °C and 0 °C for the CTmax and CTmin experiments, respectively (Supplementary Fig. 2). A layer of Vaseline was also applied to the wall of the glass cup to prevent beetles from escaping. During the whole process, we used a thermal camera (Inc., SC305, FLIR® Systems) with the software ThermaCAM Researcher Professional 2.10 to capture the pronotum area of each individual as their core temperature of CTmax or CTmin. The thermal tolerance of beetles was determined when an individual reached its critical temperature, lost coordinated leg movements, and could no longer remain in a righting position^64^. After the experiments, we returned the beetles to the walk-in growth chambers.

### Climate change simulations

We used the scenario of RCPs 8.5 for 2081–2100 (ref. ^33^) to obtain the increasing mean surface air temperature relative to the base period and directly added to the temperature pattern (data from WorldClim^31^) along the five mountain ranges. We then compared the possible distribution in fixed and varying phenological conditions by overlapping their recent thermal niche ranges in a way that projected climate change.

### Data analysis

We used non-parametric regression—LOESS^65^ with the smoothing span of 0.5—to test for differences in abundance across the five populations and five mountain ranges along the elevational and temperature gradients. To validate the reciprocal projection method of Hutchinson’s duality framework, we used a bootstrap method^66^—a random sampling technique that can help determine the sample size effect on the sampling errors—to compare the thermal niche breadth based on complete and partial data from Wulai and Mt. Hehuan. Briefly, we randomly sampled data for 1000 times for from different numbers of months. We found that randomly sampling data from the 3 months that at least one beetle appeared can generate reasonably accurate thermal niche predictions (Supplementary Fig. 1), although sampling 6 months or more generated the most accurate predictions. Thus, although we do not have year-round beetle occurrence data for the populations of Amami Oshima and Mt. Jiajin, the population density data in these populations—which we sampled four and 3 months, respectively—is enough to generate accurate temporal niche predictions.

We calculated the thermal niche breadth for different numbers of months to determine the effect of sample size on the confidence intervals of the data. We used a GLM to analyze factors influencing the breeding likelihood (i.e., burial rate) and ovarian weights of the beetles. The parents’ physical characteristics (age, pronotum width, and mass) were treated as covariates for the burying behavior experiments and the outcome (1 = burial, 0 = non-burial) was fitted as a binomial response term to test the difference in the probabilities of the breeding likelihood between photoperiods. Ovarian weight was fitted as a Gaussian response to identify the beetle’s development stage (stage 1 = just emerged, stage 2 = emerged after 7 days, and stage 3 = emerged after 14 days). A GLM was used to compare thermal niche breadth among the five populations. GLMMs were used for the fitness assays in the transplant experiments. To test for differences in the probability of breeding successfully between the two mountain ranges along the elevational and temperature gradients, the outcome of breeding (1 = success, 0 = failure) was fitted as a binomial response term. Environmental factors (elevation, daily minimum air temperature) were fitted as covariates of interest and we used the experimental location ID as a random factor. Post hoc pairwise comparisons (Tukey tests) were carried out using R package lsmeans^67^. All statistical analyses were performed in the R 3.0.2 statistical software package^68^.

### Reporting summary

Further information on research design is available in the Nature Research Reporting Summary linked to this article.

## Supplementary information

Supplementary Information

Reporting Summary

## Data Availability

The source data underlying Figs. 2, 3, 4, 5, 6, and 7 are provided as a Source Data file.

## Source data

Source Data file
