# Author Correction: Locally-adapted reproductive photoperiodism determines population vulnerability to climate change in burying beetles

**DOI:** 10.1038/s41467-020-17567-w

**Published:** 2020-07-22

**Authors:** Hsiang-Yu Tsai, Dustin R. Rubenstein, Yu-Meng Fan, Tzu-Neng Yuan, Bo-Fei Chen, Yezhong Tang, I-Ching Chen, Sheng-Feng Shen

**Affiliations:** 1 Biodiversity Research Center, Academia Sinica, Taipei, 115 Taiwan; 2 Institute of Ecology and Evolutionary Biology, College of Life Science, National Taiwan University, Taipei, 115 Taiwan; 3 Department of Ecology, Evolution and Environmental Biology and Center for Integrative Animal Behavior, Columbia University, New York, NY 10027 USA; 4 Chengdu Institute of Biology, Chinese Academy of Sciences, Chengdu, 61004 People’s Republic of China; 5 Department of Life Sciences, National Cheng Kung University, Tainan, 70101 Taiwan

**Keywords:** Climate-change ecology, Evolutionary ecology

Correction to: *Nature Communications* 10.1038/s41467-020-15208-w, published online 13 March 2020.

The original version of this Article contained an error in the “Methods”, which incorrectly omitted the “Permits” subsection as follows: To conduct the experiments, experiment permits were issued by local governments, forestry bureaus, and national parks annually in Taiwan at 2014–2018 (1054150444, 1054150442, 106000238, 1053088421, 1050273244, 106816005) MOU between two academic institutes required by National Forestry and Grassland Administration in China and the experiment permit issued by Ministry of Environment in Japan (Amami 47–82, Amami 47–83). To bring the beetles abroad back, import permits were issued by Bureau of Animal and Plant Health Inspection and Quarantine at 2018 and 2019 (1071420777 and 1061410719).

This has been corrected in both the PDF and HTML versions of the Article.

