# Supplementary material for: Locally-adapted reproductive photoperiodism determines population vulnerability to climate change in burying beetles
Source: Nat Commun. 2020 Mar 13;11:1398. doi: 10.1038/s41467-020-15208-w (PMC7069978; doi:10.1038/s41467-020-15208-w)
Supplement: Supplementary file 1 — Supplementary Information [file 41467_2020_15208_MOESM1_ESM.docx]

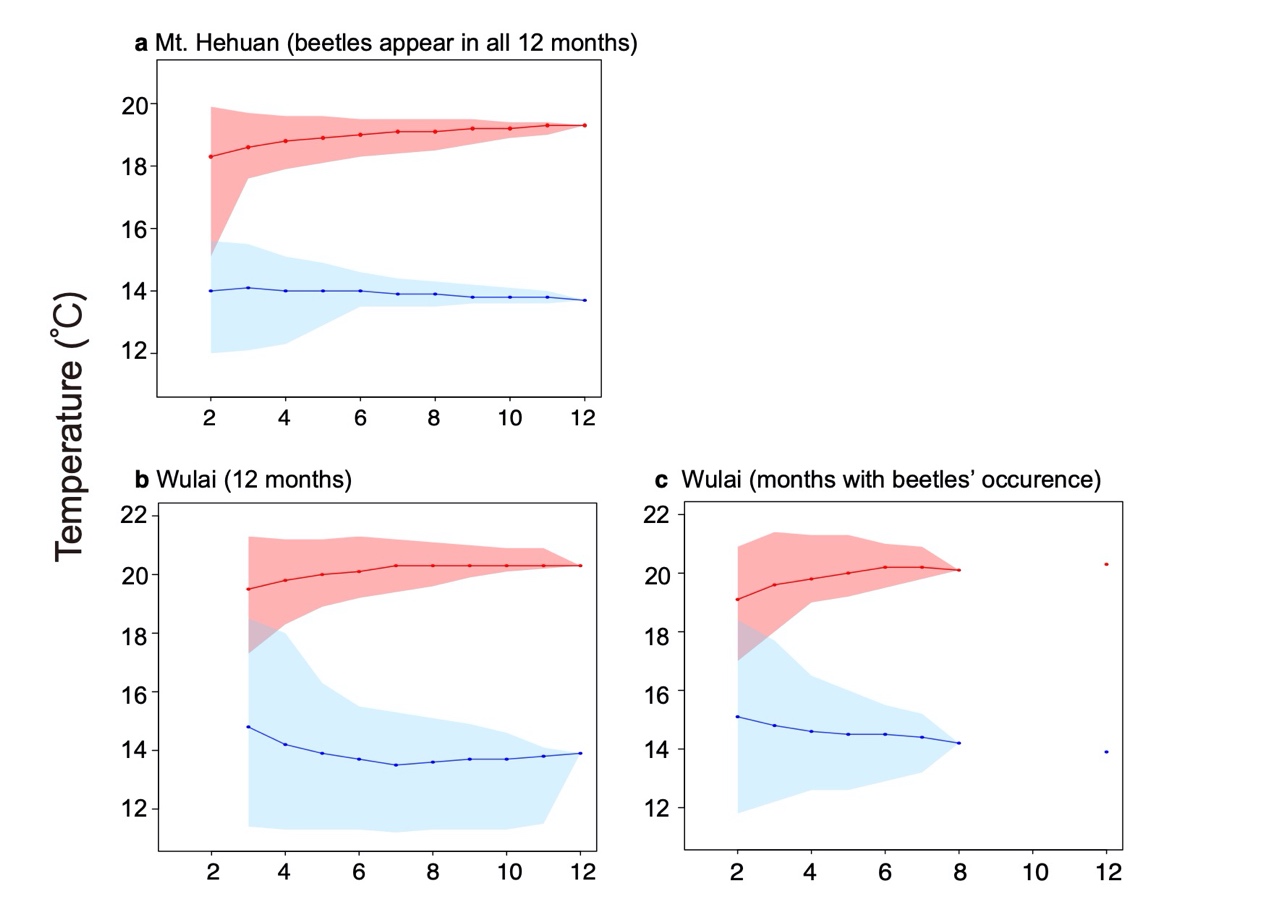


**Supplementary Fig. 1.** The potential effect of different sample sizes by bootstrapping burying beetle abundance with 1000 replicates. The average upper (red lines and red points) or lower thermal limits (blue lines and blue points) with different sample sizes (number of months) and their 95% confidence intervals (shaded regions) in a, Mt. Hehuan for 12 months, b, Wulai for 12 months and c, Wulai for eight months of beetle occurrence.

**Supplementary Fig. 2.** Thermal images taken during the a, critical thermal maximum (CTmax) and b, critical thermal minimum (CTmin) experiments. Temperature scales are shown to the right of each image.
