# Supplementary material for: Locally-adapted reproductive photoperiodism determines population vulnerability to climate change in burying beetles
Source: Nat Commun. 2020 Mar 13;11:1398. doi: 10.1038/s41467-020-15208-w (PMC7069978; doi:10.1038/s41467-020-15208-w)
Supplement: Supplementary file 2 — Reporting Summary [file 41467_2020_15208_MOESM2_ESM.pdf]

nature research

Corresponding author(s): Sheng-Feng Shen

Last updated by author(s): 2020/02/11

Reporting Summary

Nature Research wishes to improve the reproducibility of the work that we publish. This form provides structure for consistency and transparency in reporting. For further information on Nature Research policies, see Authors & Referees and the Editorial Policy Checklist .

Statistics

For all statistical analyses, confirm that the following items are present in the figure legend, table legend, main text, or Methods section.

n/a	Confirmed	
☐	☒	The exact sample size (*n*) for each experimental group/condition, given as a discrete number and unit of measurement
☐	☒	A statement on whether measurements were taken from distinct samples or whether the same sample was measured repeatedly
☐	☒	The statistical test(s) used AND whether they are one- or two-sided<br>*Only common tests should be described solely by name; describe more complex techniques in the Methods section.*
☐	☒	A description of all covariates tested
☐	☒	A description of any assumptions or corrections, such as tests of normality and adjustment for multiple comparisons
☐	☒	A full description of the statistical parameters including central tendency (e.g. means) or other basic estimates (e.g. regression coefficient) AND variation (e.g. standard deviation) or associated estimates of uncertainty (e.g. confidence intervals)
☐	☒	For null hypothesis testing, the test statistic (e.g. *F*, *t*, *r*) with confidence intervals, effect sizes, degrees of freedom and *P* value noted<br>*Give P values as exact values whenever suitable.*
☒	☐	For Bayesian analysis, information on the choice of priors and Markov chain Monte Carlo settings
☒	☐	For hierarchical and complex designs, identification of the appropriate level for tests and full reporting of outcomes
☒	☐	Estimates of effect sizes (e.g. Cohen's *d*, Pearson's *r*), indicating how they were calculated

Our web collection on statistics for biologists contains articles on many of the points above.

Software and code

Policy information about availability of computer code

Data collection	We used the software ThermaCAM Researcher Professional 2.10 to obtain the thermal tolerance data of beetles.
Data analysis	All statistical analyses were performed in the R 3.0.2 statistical software package.

For manuscripts utilizing custom algorithms or software that are central to the research but not yet described in published literature, software must be made available to editors/reviewers. We strongly encourage code deposition in a community repository (e.g. GitHub). See the Nature Research guidelines for submitting code & software for further information.

Data

Policy information about availability of data

All manuscripts must include a data availability statement. This statement should provide the following information, where applicable:
- Accession codes, unique identifiers, or web links for publicly available datasets
- A list of figures that have associated raw data
- A description of any restrictions on data availability

The source data underlying Figs. 2-7 are provided as a Source Data file.

Field-specific reporting

Please select the one below that is the best fit for your research. If you are not sure, read the appropriate sections before making your selection.

☐ Life sciences ☐ Behavioural & social sciences ☒ Ecological, evolutionary & environmental sciences

For a reference copy of the document with all sections, see nature.com/documents/nr-reporting-summary-flat.pdf

Ecological, evolutionary & environmental sciences study design

All studies must disclose on these points even when the disclosure is negative.

Study description	It is a phenological and physiological evolution finding among five populations of burying beetles in a Hutchinson's duality concept, we further assessed their vulnerability toward climate change in each population. density survey, reciprocal transplant experiments in Mt. Hehuan and Wulai (summer in Wulai: 120 replicates; summer in Mt. Hehuan: 93 replicates; winter in Wulai: 78 replicates; winter in Mt. Hehuan: 83 replicates), common garden experiments under different photoperiod (Wulai population: 126 replicates; Amami population: 41 replicates; Mt. Hehuan population: 50 replicates; Mt. Lala: 32 replicates; Mt. Jiajin: 49 replicates.) and under different temperature (Wulai: 24 replicates, Amami Oshima: 88 replicates, Mt. Lala: 44 replicates, Mt. Hehuan: 70 replicates, Mt. Jiajin: 18 replicates), ovary dissection experiments (long photoperiod condition: 55 replicates; short photoperiod condition: 44 replicates.) and thermal tolerance experiments (Wulai population: 120 replicates; Amami population: 83 replicates; Mt. Hehuan population: 219 replicates; Mt. Lala: 60 replicates; Mt. Jiajin: 40 replicates).
Research sample	Nicrophorus nepalensis from five populations: Mt. Hehaun, Taiwan, Mt. Lala, Taiwan, Wulai, Taiwan, Amami Oshima, Japan and Mt. Jiajin, China. We used the beetles which aged 2 to 3 weeks after emergence. No specific selection of individuals during field collection in each population but equal amounts of males and females were used in the breeding experiments.
Sampling strategy	To prevent type I and type II error, we set the significance level as 0.05, effect size as 0.8, and the statistical power as 0.8. We then got the sample size should be at least 25.
Data collection	For the density survey, burying beetles were collected using hanging pitfall traps baited with rotting pork at each sites. The pitfall traps were checked in the morning on the fourth day of the experiment. Each beetle's pronotum was measured to the nearest 0.01 mm and sex was determined by the markings on the clypeus. The air temperature at every site was measured using iButton® devices that were placed approximately 120 cm above the ground within a T-shaped PVC pipe to prevent direct exposure to the sun<br>For the reciprocal transplant experiments, we chose summer and winter in Mt. Hehuan and Wulai in Taiwan and the three sites on each mountain that the highest beetle abundance according in our field surveys. We conducted the experiments in which lab strains from either population were transplanted to the foreign mountain range (i.e. the lab strain individuals originating from the Wulai population were transplanted to Mt. Hehuan, and vice versa) and transplanted to native mountain range as the control (i.e. the lab strain individuals originating from the Wulai or Mt. Hehuan population were transplanted to Wulai or Mt. Hehuan, respectively). In each trial, we placed a male and a female with a mouse carcass (75 ± 7.5 g) in the breeding pot, which was covered by a gauze web and buried in the soil. A 75 g rat carcass was placed on the soil and covered with a 21 × 21 × 21 cm iron cage with a mesh size of 2 × 2 cm to prevent vertebrate scavengers from accessing the carcass. After two weeks, we would check the trail to see if they bury and breed or not. H.-Y. T., Y.-M. F., T.-N. Y., B.-F. C., Y.T. and S.-F.S. performed the field studies.<br>For the common garden experiments under different photoperiod , we conducted a solitary pairing experiment in two photoperiodic regimes: long- (10 hrs Dark : 14 hrs Light) and short-day conditions (14 hrs Dark : 10 hrs Light). The temperature and humidity were set to the same conditions in both photoperiodic regimes, as described above. All larvae from a pair of beetles were separated into the two photoperiodic conditions immediately at the time of dispersal. We used sexually mature beetles aged 2 to 3 weeks after emergence. H.-Y. T. and Y.-M. F. conducted the lab experiments.<br>For the common garden experiments under different temperature, we conducted pair breeding experiments in three average temperature condition: 12℃, 16℃, and 20℃. The humidity was set to the same conditions in all temperature conditions (RH: 83-100%). The photoperiod regime was set to the same as short-day conditions (14 hrs Dark : 10 hrs Light) for the Wulai, Amami Oshima, Mt. Lala, and Mt. Hehuan population, but to long-day conditions (10 hrs Dark : 14 hrs Light) for the Mt. Jiajin population since these beetles can only breed under long-day conditions. All larvae from a pair of beetles were separated into the two photoperiodic conditions immediately at the time of dispersal. We used sexually mature beetles aged 2 to 3 weeks after emergence.<br>For the ovary dissection experiments, we dissected and quantified ovarian weight at three-time points: on days 0, 7, and 14 after emergence. The general dissection protocol followed the method described by Wilson and Knollenberg. Briefly, we stored the beetles in ice for one hour to kill them. We began by measuring their body weight and cutting down their abdomens. Ovaries were then dissected in Ringer's solution. Next, we removed the spermatheca and accessory glands and immediately examined the wet weights of each organ. H.-Y. T. and Y.-M. F. conducted the lab experiments.<br>For the thermal tolerance experiments, we first measured burying beetles' pronotum and transferred them to separate glass cups (200 mL, with lid) to calm down. This process usually took 1 hour at ambient temperature, which was the initial temperature of the thermal trail. The cup with the beetle was then submerged into either a 50°C or –10°C water bath to test its upper or lower thermal limit. Thermal limit was determined when an individual reached its critical temperature, lost coordinated leg movements, and could no longer remain in a roosting position. When this occurred, we immediately took them out of the cup to record their body temperature by a thermal camera (InfraCAM, FLIR Systems). Finally, we used ThermaCAM Researcher Pro 2.10 software (FLIR Systems) to capture the pronotum area of each individual as their core temperature of critical thermal maximum (CTmax) and minimum (CTmin). H.-Y. T. and Y.-M. F. conducted the lab experiments.
Timing and spatial scale	Time scale:<br>We did the experiments for at most three months at each site to generate reasonably accurate thermal niche predictions.<br>We did density survey monthly for the monthly survey at Mt. Hehaun, Taiwan (January 2016 to May 2018), Mt. Lala, Taiwan (February-April, August and November 2017; February, June and July 2018), Wulai, Taiwan (January 2016 to May 2018), Amami Oshima, Japan (February 2015; April 2016; March-April 2018) and Mt. Jiajin, China (June-August 2017,January June-August 2018 and November 2019).<br>Spatial scale:<br>We chose the experiments sites from the lowest elevation to the highest in each habitat.<br>Wulai in Taiwan, with natural habitats ranging from 200m (121°51'E, 24°83'N) to 900m (121°54'E 24°85'N) above sea level (hereafter a.s.l.) and Amami Oshima in Japan, ranging from 60m (129°27'E, 28°32'N) to 700m (129°32'E 28°30'N) a.s.l—one intermediate height mountain range—Mt. Lala in Taiwan, ranging from 400m (121°51'E, 24°60'N) to 2000m (121°43'E, 24°73'N) a.s.l.—and two high mountain ranges—Mt. Hehuan in Taiwan, ranging from 500m (121°00'E 23°98'N) to 3200m (121°27'E, 24°13'N) and Mt. Jiajin in

	China, ranging from 800m (102°84'E, 30°23'N) to 4100m (102°68'E 30°86'N) a.s.l .
Data exclusions	The result that beetles were dead during the experiments would be excluded to prevent the impact on the breeding result due to the beetles' death.
Reproducibility	Both our field and lab experiments were based on large sample sizes but we did not repeat our experiments.
Randomization	We randomly picked the beetles from different nests to conduct the breeding experiments in common garden, the ovary dissection experiments, thermal tolerance experiments and the transplant experiments.
Blinding	While doing experiments, we picked the populations randomly to conduct all the experiments. We didn't expect any difference among populations while first conducting the breeding experiments and followed the protocol strictly. Thus, we gave them the equal environment and condition blindly.<br>During data analysis, to prevent any bias toward the result, we randomly numbered our experimental samples regardless the treatment and test the relationship between number and the response vector.

Did the study involve field work? ☒ Yes ☐ No

Field work, collection and transport

Field conditions	Beetles samples were mostly collected in natural forest in all weather conditions.
Location	Wulai in Taiwan, with natural habitats ranging from 200m (121°51'E, 24°83'N) to 900m (121°54'E 24°85'N) above sea level (hereafter a.s.l.) and Amami Oshima in Japan, ranging from 60m (129°27'E, 28°32'N) to 700m (129°32'E 28°30'N) a.s.l—one intermediate height mountain range—Mt. Lala in Taiwan, ranging from 400m (121°51'E, 24°60'N) to 2000m (121°43'E, 24°73'N) a.s.l.—and two high mountain ranges—Mt. Hehuan in Taiwan, ranging from 500m (121°00'E 23°98'N) to 3200m (121°27'E, 24° 13'N) and Mt. Jiajin in China, ranging from 800m (102°84'E, 30°23'N) to 4100m (102°68'E 30°86'N) a.s.l .
Access and import/export	We drove to all the plots in each site to conduct the experiments. To conduct the experiments, we applied the experiment permits required by local governments, forestry bureaus and national parks annually in Taiwan at 2014-2018, MOU between two academic institutes required by National Forestry and Grassland Administration in China and the experiment permit required by Ministry of Environment in Japan.<br>To bring the beetles abroad back, we have apply for the import permit required by Bureau of Animal and Plant Health Inspection and Quarantine at 2018 and 2019.
Disturbance	We collect samples only once a month at the same location to minimized the disturbance frequency and impacted regions.

Reporting for specific materials, systems and methods

We require information from authors about some types of materials, experimental systems and methods used in many studies. Here, indicate whether each material, system or method listed is relevant to your study. If you are not sure if a list item applies to your research, read the appropriate section before selecting a response.

Materials & experimental systems

n/a	Involved in the study	
☒	☐	Antibodies
☒	☐	Eukaryotic cell lines
☒	☐	Palaeontology
☐	☒	Animals and other organisms
☒	☐	Human research participants
☒	☐	Clinical data

Methods

n/a	Involved in the study	
☒	☐	ChIP-seq
☒	☐	Flow cytometry
☒	☐	MRI-based neuroimaging

Animals and other organisms

Policy information about studies involving animals; ARRIVE guidelines recommended for reporting animal research

Laboratory animals	Nicrophorus nepalensis from five populations: Mt. Hehaun, Taiwan, Mt. Lala, Taiwan, Wulai, Taiwan, Amami Oshima, Japan and Mt. Jiajin, China. We used the beetles which aged 2 to 3 weeks after emergence.
Wild animals	Nicrophorus nepalensis from five populations: Mt. Hehaun, Taiwan, Mt. Lala, Taiwan, Wulai, Taiwan, Amami Oshima, Japan and Mt. Jiajin, China. We used the hanging pitfall traps baited with rotting pork at each sites. The pitfall traps were checked in the morning on the fourth day of the experiment. After measuring beetle's pronotum and determining the sex by the markings on the clypeus, we released the beetles right after recording all the beetles' details. We only brought 2 males and 2 females of beetles in each plot back to the lab to make sure we didn't have a strong influence on the population density in the field. All wild-caught beetles were transported to the laboratory and allowed to reproduce in captivity.
Field-collected samples	Beetles were housed individually in 320 ml transparent plastic cups and fed superworms (Zophobas morio) weekly if they were kept for more than three days before the experiment. Beetles captured in the relatively short photoperiod season would be kept

	in the short day conditions (14hrs Dark : 10 hrs Light) and beetles captured in the relatively short photoperiod season would be kept in the long day conditions (10hrs Dark : 14 hrs Light). The temperature and humidity were set to the same conditions in both photoperiodic regimes (daily temperature cycles between 19°C at noon and 13°C at midnight; RH: 83-100%), which imitated natural conditions at 2100 m elevation on Mt. Hehaun. After study, we would stored the beetles in the -80 refrigerator.
Ethics oversight	No formal ethical approval was required except sampling permit because agencies in Taiwan (and many other places) only review vertebrates studies.

Note that full information on the approval of the study protocol must also be provided in the manuscript.
